# Does smoking impact trust in physicians and satisfaction with the health system in China?

**DOI:** 10.18332/tid/131626

**Published:** 2021-01-25

**Authors:** Changle Li, Zhengzhong Mao, Gang He, Qitu Hu

**Affiliations:** 1 Department of Health Economics, School of Health Management, Inner Mongolia Medical University, Hohhot, China; 2 Huaxi School of Public Health, Sichuan University, Chengdu, China; 3 General Affairs Section, Chifeng Municipal Hospital, Chifeng, China; 4 Department of General Psychology, College of Basic Medicine, Inner Mongolia Medical University, Hohhot, China

**Keywords:** smoking, trust in physicians, satisfaction, health system, China

## Abstract

**INTRODUCTION:**

Trust and satisfaction play vital roles in how smokers react to smoking cessation treatment delivered by physicians. This paper aims to ascertain whether smoking status and pack-years of smoking affect trust in physicians and satisfaction with the health system in China.

**METHODS:**

The current study used the ordered probit model to evaluate how smoking status affects trust in physicians and satisfaction with the health system in China. Data from the China Family Panel Studies (CFPS) 2018 were used for the analysis, and the final sample consisted of 29500 adults. The CFPS is a nationally representative, comprehensive, high-quality, biennial longitudinal survey of Chinese communities, families, and individuals. The survey was conducted in 25 provinces and their administrative equivalents. The population of 25 provinces represents 95% of the total population in Mainland China.

**RESULTS:**

According to the ordered probit model, the results showed that current smokers were significantly negatively associated with trust in physicians, and more cigarette smoking was associated with decreased trust in physicians. Moreover, current smokers were also significantly negatively related to satisfaction with the health system.

**CONCLUSIONS:**

The present study found that current smokers would be more likely to rate trust in physicians lower, and less likely to rate greater satisfaction with the health system, than never smokers. These results may have important implications for regaining trust in physicians from smokers and supporting health-system reform for tobacco treatment.

## INTRODUCTION

In 2018, an estimated 26.6% of Chinese adults were current smokers, more in men (50.5%) than in women (2.1%), implying that the number of current smokers was more than 300 million^1^. Even though smoking has proven to be a significant cause of diseases such as cardiovascular disease, chronic obstructive lung disease, and lung cancer, only 19.8% of current smokers tried to quit smoking within one year. Among those people, 8.4 % of smokers used pharmacotherapies and counseling for smoking cessation^1,2^. Trust and satisfaction play essential roles in how smokers react to smoking cessation treatment delivered by physicians^3,4^. Trust in physicians increases healthcare utilization^5^. Increasing patient trust of physicians is likely to increase the use of preventive services^6^. Moreover, patient satisfaction is significantly associated with the performance of preventive services^7^. Therefore, it is necessary to examine trust in physicians and satisfaction in healthcare among smokers to promote smokers’ utilization of preventive care and adherence to prescribed care.

Although previous studies have analyzed the relationship between smoking status and trust, the relationship between smoking status and trust is complicated. Rural Medicaid-insured smokers have a high level of trust in their healthcare provider^8^. Smokers have a significantly higher level of trust in cancer information obtained from their physicians^9^. Interestingly, some studies found that current smokers report a low level of trust in physicians than never smokers^10^, and daily smokers are less likely to trust physicians compared to never smokers^11,12^. In addition, lower satisfaction with healthcare providers is associated with being a current smoker^13^, but smokers who receive smoking cessation treatment are associated with higher satisfaction with their healthcare^14,15^.

As the above relationship has not been examined in China, a national survey was used to ascertain trust in physicians and satisfaction with the health system among Chinese smokers. This paper aims to ascertain whether smoking status and pack-years of smoking affect trust in physicians and satisfaction with the health system in China.

## METHODS

### Data source

The data used in this study were obtained from the China Family Panel Studies 2018 (CFPS 2018), launched by the Institute of Social Science Survey of Peking University. The CFPS is a nationally representative, comprehensive, high-quality, biennial longitudinal survey of Chinese communities, families, and individuals. The survey was conducted in 25 provinces and their administrative equivalents. The population of 25 provinces represents 95% of the total population in Mainland China. The CFPS uses multistage probability proportional-to-size sampling and includes community, family, adult, and child, questionnaires. The CFPS implemented the first wave survey in 2010 and four waves of full sample follow-up surveys in 2012, 2014, 2016, and 2018. A detailed description of the CFPS is provided in previous studies^16,17^. The CFPS 2018 included 32539 adults aged ≥15 years. Only adults who completed the questionnaire by themselves were selected from the dataset, and the final sample consisted of 29500 adults.

### Measures

Trust in physicians characterizes a relationship between the patient and his/her physician. However, unlike in many developed countries, the idea of ‘my physician’ is somewhat unusual among Chinese people due to the lack of contracted family physicians in the primary care system. General trust in physicians was set as an ordinal dependent variable with eleven response categories based on a CPFS question asking respondents to indicate their overall trust in physicians on a scale from 0 (completely distrustful) to 10 (completely trustworthy).

Satisfaction with the health system was also set as an ordinal dependent variable. This variable takes values between 0 and 10; with 0 completely unsatisfied, and 10 completely satisfied. The CFPS question evaluating this variable was: ‘How would you rate the severity of China's healthcare problem?’; with 0 ‘least severe’, and 10 ‘most severe’. The severity score for the healthcare problem measures public satisfaction with the health system^18^. The current study recoded the value of 10 for ‘most severe’ to 0 for ‘completely unsatisfied’; and 0 for ‘least severe’ to 10 for ‘completely satisfied’.

All respondents were divided into three mutually exclusive smoking-status groups based on the last wave of the CFPS: non-smokers, current smokers, and ex-smokers. The CFPS 2018 question asked: ‘Have you smoked in the past month?’. Responses were ‘Yes’ or ‘No’. If the respondent answered ‘Yes’, the respondent was categorized as a current smoker. The respondent who answered ‘No’ was then asked: ‘Have you ever smoked?’. Responses were ‘Yes’ or ‘No’. If the respondent answered ‘Yes’, the respondent was considered as an ex-smoker. If the respondent answered ‘No’ to both questions, the respondent was categorized as a non-smoker. Moreover, all current smokers reported the number of cigarettes smoked per day and the number of years of smoking. Pack-years of smoking were then calculated by multiplying the number of packs of cigarettes smoked per day by the number of years of smoking; ex-smokers and non-smokers were assigned a score of 0.

The control variables were selected based on a literature review to identify factors that may affect public trust in physicians and satisfaction with the health system. These variables were classified into the following four categories: 1) sociodemographic characteristics such as age, sex, educational level, marital status, urban residency, medical insurance, household income, and employment; 2) health status, self-rated health and smoking-related chronic conditions; 3) drinking; and 4) healthcare utilization.

An adult with smoking-related chronic diseases was defined as a respondent who self-reported to have been diagnosed with cancer, cardiovascular disease, lung diseases and/or diabetes between 2010 and 2018 based on five waves of data from the CFPS. Healthcare utilization was set as a dummy variable equal to 1 if the individual self-reported being hospitalized in the past 12 months and 0 otherwise, based on the CFPS question: ‘Have you been hospitalized in the past twelve months?’. In empirical modeling, to capture differences in frequency of visits, inpatient-care utilization can be used as a proxy. Table 1 describes all the variables in this study.

**Table 1:** Definitions of variables, evaluations, and sociodemographic characteristics, China Family Panel Studies 2018 (N=29500)

Variables	Description	Mean (SD) or %
**Dependent variables**		
TIP	Trust in physicians. An ordinal variable with eleven response categories from 0 (completely distrustful) to 10 (completely trustworthy)	6.74 (2.38)
SHS	Satisfaction with the health system. An ordinal variable with five response categories from 0 (completely unsatisfied) to 10 (completely satisfied)	3.34 (2.70)
**Independent variables**		
**Smoking status**		
Current smoker	Coded: 1 if the individual currently smokes cigarettes; 0 otherwise	28.81
Ex-smoker	Coded: 1 if the individual has quit smoking; 0 otherwise	2.28
Never smoker	Coded: 1 if the individual has never smoked; 0 otherwise	68.91
**Pack-years**	One pack-year is 20 cigarettes smoked per day for one year	11.19 (24.90)
**Age** (years)		
15–24	Coded: 1 if the individual is aged 15–24 years; 0 otherwise	10.97
25–64	Coded: 1 if the individual is aged 25–64 years; 0 otherwise	71.96
≥65	Coded: 1 if the individual is aged ≥65 years; 0 otherwise	17.07
**Male**	Coded: 1 if the individual is male; 0 for female	49.57
**Educational level**		
Illiterate	Coded: 1 if the individual is illiterate or semi-literate; 0 otherwise	21.77
Elementary school	Coded: 1 if the individual attends elementary school; 0 otherwise	19.78
Middle school	Coded: 1 if the individual graduated only from middle school; 0 otherwise	29.93
High school	Coded: 1 if the individual graduated only from high school; 0 otherwise	16.24
Above three-year college	1 if the individual graduated from above three-year college; 0 otherwise	12.29
**Married**	Coded: 1 if the individual is married; 0 otherwise	78.49
**Urban residency**	Coded: 1 if the individual is an urban resident; 0 for a rural resident	50.48
**Medical insurance**		
GMI	Coded: 1 if the individual has Government Medical Insurance; 0 otherwise	2.34
UEMI	Coded: 1 if the individual has Urban Employee Medical Insurance; 0 otherwise	14.57
URMI	Coded: 1 if the individual has Urban Resident Medical Insurance; 0 otherwise	8.49
NRCMI	Coded: 1 if the individual has New Rural Cooperative Medical Insurance; 0 otherwise	65.16
SI	Coded: 1 if the individual has supplementary medical insurance; 0 otherwise	0.44
NoI	Coded: 1 if the individual does not have medical insurance; 0 otherwise	11.34
**Household income**	Net household income (×10000 RMB)	6.20 (7.58)
**Employment**		
Agricultural	Coded: 1 if the individual works in agriculture; 0 otherwise	31.85
Waged	Coded: 1 if the individual has an employer; 0 otherwise	34.13
Self-employed	Coded:1if theindividualworksforhimself/herself; 0 otherwise	8.34
Other	Coded:1if theindividualisatemporaryworker,retired,unemployed, or student;0otherwise	25.67
**Health status**		
Poor	Coded: 1 if the individual reports health status to be poor; 0 otherwise	16.29
Fair	Coded:1 iftheindividual reports health status to be fair; 0 otherwise	13.09
Good	Coded: 1 if the individual reports health status to be good, very good, or excellent; 0 otherwise	70.62
**Smoking-related chronic diseases**	Coded: 1 if the individual has had doctor-diagnosed smoking-related chronic diseases; 0 otherwise	16.78
**Drinking**	Coded: 1 if the individual reports drinking alcohol at least three times a week in the past month; 0 otherwise	14.9
**Healthcare utilization**	Coded: 1 if the individual is hospitalized in the past twelve months; 0 otherwise	13.11

RMB: 1000 Chinese Renminbi about 150 US$.

### Statistical analysis

To detect smoking status affecting trust in physicians in China, the current study used the ordered probit model. The ordered probit model is based on a latent regression and is defined as follows:

y*=x' a+ε

where *x*' is a vector of independent variables including smoking status or pack-years of smoking, sociodemographic characteristics, health status, drinking, and healthcare utilization, a is the coefficient vector, y* is an unobserved latent variable linked to the observed ordinal response categories of ‘Trust in physicians’ (TIP):

$$\mathrm{TIP}=\left[\begin{array}{cc} 0, & if\;y^{*}\;\leq \mu_{0}\\ 1,\; & if\mu_{0}\text{ < }y^{*}\;\leq \;\mu_{1}\\ & .\\ & .\\ & .\\ 9, & \text{if }\mu_{8}\text{ < }y^{*}\;\leq \;\mu_{9}\\ 10,\; & if\;\mu_{9}\text{ < }y^{*} \end{array}\right]$$

The unknown parameter *μ* is to be estimated with *a*. We assume that *ε* is normally distributed across observations and normalize the mean and variance of *ε* to 0 and 1, respectively. We then have the following probabilities:

*Prob(TIP*=0*│x)=Φ(-x' β)*

*Prob(TIP*=1│x*)=Φ(u_1_-x' β)-Φ(-x' β)*

⋮

*Prob(TIP*=10│x*)=1-Φ(u_9_-x' β)*

In order for all the probabilities to be positive, we must have 0<*u*_1_<*u*_2_<···<*u*_9_. The ordered probit model was estimated using maximum likelihood in the statistical software package STATA 14.0. The present study also employed the ordered probit model to explore smoking status affecting satisfaction with the health system in China.

## RESULTS

**Figure 1:**
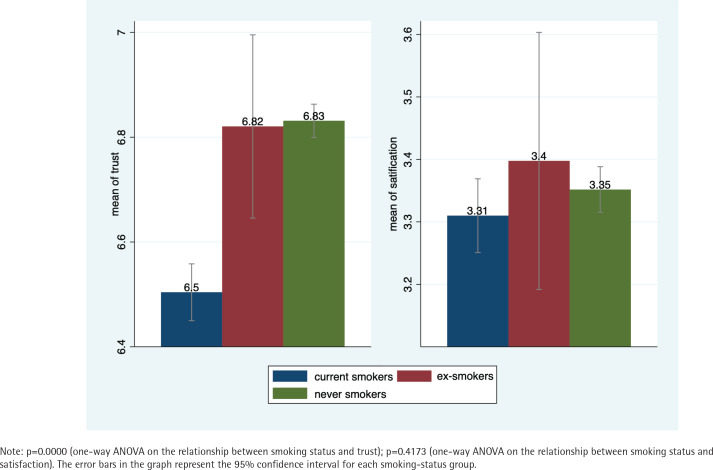
The mean trust score and satisfaction score by smoking status

Table 1 reports the characteristics of the sample. Nearly 30% of the study population were current smokers. Only about 2% of the study population were ex-smokers. The mean trust score and satisfaction score were 6.74 and 3.34, respectively. Figure 1 presents the mean trust score and satisfaction score by smoking status. Compared to never smokers and ex-smokers, current smokers had a low level of trust in physicians and low satisfaction with the health system. One-way ANOVA showed significant differences in trust in physicians across smoking status. However, there were no significant satisfaction differences across smoking status.

Table 2 shows the results of smoking status and pack-years affecting trust in physicians. The coefficient estimates for Model 1 are shown in column (i) of Table 2 for the categories of smoking status. When the individual was a current smoker, he/she would be more likely to rate his/her trust in physicians lower than a never smoker (Coefficient = -0.0609; 95% CI: -0.0938, -0.0280). Ex-smokers were more likely to rate trust in physicians higher than never smokers, but not statistically significant (Coefficient = 0.0470; 95% CI: -0.0334, 0.1280). The coefficient estimates for Model 2 are shown in column (iii) of Table 2 for pack-years categories. More cigarette smoking, measured in pack-years, was associated with decreased trust in physicians (Coefficient = -0.000974; 95% CI: -0.001510, -0.000439).

**Table 2:** Ordered probit regression analysis on trust in physicians

*Variable*	*Model 1*		*Model 2*	
	*(i)*	*(ii)*	*(iii)*	*(iv)*
	*Coefficient (95% CI)*	*SE*	*Coefficient (95% CI)*	*SE*
**Smoking status**				
Current smoker	-0.0609** (-0.0938, -0.0280)	0.0168	-	
Ex-smoker	0.0470 (-0.0334, 0.1280)	0.0411	-	
Never smoker	Ref.		-	
**Pack-years**	-		-0.000974** (-0.001510, -0.000439)	0.000273
**Age**				
15–24	Ref.		Ref.	
25–64	-0.1590** (-0.2080, -0.1100)	0.0251	-0.1580** (-0.2070, -0.1090)	0.0251
≥65	-0.0273 (-0.0845, 0.0298)	0.0292	-0.0218 (-0.0792, 0.0356)	0.0293
**Male**	-0.0692** (-0.0998, -0.0386)	0.0156	-0.0792** (-0.107, -0.0518)	
**Educational level**				
Illiterate	Ref.		Ref.	
Elementary school	-0.0902** (-0.1280, -0.0523)	0.0193	-0.0887** (-0.1270, -0.0508)	0.0193
Middle school	-0.1020** (-0.1390, -0.0643)	0.0192	-0.1020** (-0.1390, -0.0642)	0.0192
High school	-0.1090** (-0.1540, -0.0636)	0.0231	-0.1090** (-0.1540, -0.0636)	0.0231
Above three-year college	0.00258 (-0.0511, 0.0563)	0.0274	0.00225 (-0.0515, 0.0560)	0.0274
**Married**	-0.00965 (-0.0451, 0.0258)	0.0181	-0.00807 (-0.0436, 0.0274)	0.0181
**Urban residency**	-0.1210** (-0.1480, -0.0938)	0.0138	-0.1200** (-0.1470, -0.0933)	0.0138
**Medical insurance**				
GMI	-0.0157 (-0.1030, 0.0721)	0.0448	-0.0124 (-0.1000, 0.0753)	0.0448
UEMI	0.0563* (0.0029, 0.1100)	0.0273	0.0576* (0.0042, 0.1110)	0.0273
URMI	0.0510 (-0.0062, 0.1080)	0.0292	0.0524 (-0.0048, 0.1100)	0.0292
NRCMI	0.1220** (0.0790, 0.1650)	0.0219	0.1220** (0.0794, 0.1650)	0.0219
SI	0.1030 (-0.0777, 0.2840)	0.0923	0.1050 (-0.0758, 0.2860)	0.0923
NoI	Ref.		Ref.	
**Household income**	0.00140 (-0.00026, 0.00306)	0.00085	0.00142 (-0.00024, 0.00308)	0.00085
**Employment**				
Agricultural	0.0929** (0.0575, 0.1280)	0.0181	0.0923** (0.0569, 0.128)	0.0181
Waged	-0.0582** (-0.0928, -0.0236)	0.0177	-0.0620** (-0.0966, -0.0274)	0.0176
Self-employed	-0.1160** (-0.1650, -0.0665)	0.0251	-0.1180** (-0.1670, -0.0688)	0.0251
Other	Ref.		Ref.	
**Health status**				
Poor	-0.0061 (-0.0508, 0.0387)	0.0228	-0.0071 (-0.0519, 0.0376)	0.0228
Fair	Ref.		Ref.	
Good	0.1090** (0.0725, 0.1450)	0.0184	0.1070** (0.0711, 0.1430)	0.0184
**Smoking-related chronic diseases**	-0.0224 (-0.0571, 0.0123)	0.0177	-0.0212 (-0.0559, 0.0135)	0.0177
**Drinking**	-0.0396* (-0.0751, -0.0041)	0.0181	-0.0396* (-0.0751, 0.0040)	0.0181
**Healthcare utilization**	0.0478* (0.0107, 0.0848)	0.0189	0.0479* (0.0109, 0.0849)	0.0189
Number of observations	29500		29500	

** Statistical significance at 1% level

* Statistical significance at 5% level.

Table 3 shows the results of smoking status and pack-years affecting satisfaction with the health system. Column (i) reports the coefficient estimates for Model 1 in which smoking-status variables are included. Individuals who currently smoke cigarettes were less likely to rate greater satisfaction with the health system compared with those who never smoked (Coefficient = -0.0737; 95% CI: -0.1060, -0.0405). Column (iii) reports the coefficient estimates for Model 2 in which pack-years are included. More cigarette smoking, measured in pack-years, was associated with lower satisfaction with the health system, but not statistically significant (Coefficient = 0.000122; 95% CI: -0.000415, 0.000659).

**Table 3:** Ordered probit regression analysis on satisfaction with the health system

*Variable*	*Model 1*		*Model 2*	
	*(i)*	*(ii)*	*(iii)*	*(iv)*
	*Coefficient (95% CI)*	*SE*	*Coefficient (95% CI)*	*SE*
**Smoking status**				
Current smoker	-0.0737** (-0.1060, -0.0405)	0.0169	-	
Ex-smoker	-0.0334 (-0.1140, 0.0477)	0.0414	-	
Never smoker	Ref.		-	
**Pack-years**	-		0.000122 (-0.000415, 0.000659)	0.000274
**Age**				
15–24	Ref.		Ref.	
25–64	-0.1940** (-0.2430, -0.1440)	0.0252	-0.2030** (-0.2520, -0.1530)	0.0252
≥65	0.00724 (-0.0500, 0.0645)	0.0292	0.000886 (-0.0566, 0.0583)	0.0293
**Male**	0.0908** (0.0599, 0.1220)	0.0157	0.0513** (0.0236, 0.0789)	0.0141
**Educational level**				
Illiterate	Ref.		Ref.	
Elementary school	-0.1220** (-0.1600, -0.0843)	0.0193	-0.1210** (-0.1590, -0.0833)	0.0193
Middle school	-0.2180** (-0.2550, -0.1810)	0.0192	-0.2160** (-0.2530, -0.1780)	0.0192
High school	-0.3240** (-0.3700, -0.2790)	0.0232	-0.3210** (-0.3670, -0.2760)	0.0233
Above three-year college	-0.4380** (-0.4920, -0.3830)	0.0278	-0.4290** (-0.4840, -0.3750)	0.0278
**Married**	-0.0027 (-0.0385, 0.0330)	0.0182	-0.0019 (-0.0377, 0.0338)	0.0182
**Urban residency**	-0.0126 (-0.0399, 0.0146)	0.0139	-0.0122 (-0.0395, 0.0150)	0.0139
**Medical insurance**				
GMI	-0.0529 (-0.142, 0.0365)	0.0456	-0.0510 (-0.140, 0.0383)	0.0456
UEMI	-0.0618* (-0.1160, -0.0076)	0.0276	-0.0608* (-0.1150, -0.0066)	0.0276
URMI	-0.0085 (-0.0664, 0.0494)	0.0295	-0.0092 (-0.0671, 0.0487)	0.0295
NRCMI	0.0252 (-0.0182, 0.0685)	0.0221	0.0250 (-0.0183, 0.0684)	0.0221
SI	0.0121 (-0.1700, 0.1950)	0.0931	0.0131 (-0.1690, 0.1960)	0.0931
NoI	Ref.		Ref.	
**Household income**	0.00126 (-0.00042, 0.00295)	0.00086	0.00130 (-0.00038, 0.00299)	0.00086
**Employment**				
Agricultural	0.0350 (-0.000469, 0.0704)	0.0181	0.0322 (-0.00324, 0.0676)	0.0181
Waged	-0.0699** (-0.1050, -0.0350)	0.0178	-0.0741** (-0.1090, -0.0392)	0.0178
Self-employed	-0.1070** (-0.1570, -0.0570)	0.0255	-0.1090** (-0.1590, -0.0588)	0.0254
Other	Ref.		Ref.	
**Health status**				
Poor	-0.1210** (-0.1660, -0.0759)	0.0230	-0.1210** (-0.1660, -0.0759)	0.0230
Fair	Ref.		Ref.	
Good	-0.0321 (-0.0683, 0.0042)	0.0185	-0.0329 (-0.0691, 0.0034)	0.0185
**Smoking-related chronic diseases**	0.0040 (-0.0308, 0.0389)	0.0178	0.00518 (-0.0297, 0.0400)	0.0178
**Drinking**	0.0299 (-0.0059, 0.0658)	0.0183	0.0197 (-0.0162, 0.0556)	0.0183
**Healthcare utilization**	0.0337 (-0.0034, 0.0709)	0.0190	0.0351 (-0.0021, 0.0722)	0.0190
Number of observations	29500		29500	

** Statistical significance at 1% level

* Statistical significance at 5% level

Table 4 presents the results of the average marginal effects of smoking status on trust in physicians and satisfaction with the health system. Trust in physicians or satisfaction with the health system had a significant negative coefficient concerning current smokers, i.e. current smokers were less likely to rate complete trust in physicians compared to those who never smoked. Moreover, current smokers were less likely to rate completely satisfied with the health system than never smokers. In particular, ex-smokers were more likely to rate complete trust in physicians than never smokers, but not statistically significant.

**Table 4:** Estimated marginal effect of smoking status on trust in physicians and satisfaction with the health system

*Smoking status*	*Trust in physicians*	*Marginal effect (95% CI)*	*Satisfaction with the health system*	*Marginal effect (95% CI)*
Current smoker	Completely trustworthy	-0.0146** (-0.0223, -0.00681)	Completely satisfied	-0.00547** (-0.00788, -0.00306)
Ex-smoker	Completely trustworthy	0.0117 (-0.00872, 0.0321)	Completely satisfied	-0.00247 (-0.00831, 0.00337)
Never smoker		Ref.		Ref.

Adjusted for age, sex, educational level, marital status, urban residency, medical insurance, household income, employment, self-rated health, smoking-related chronic diseases, drinking, and healthcare utilization.

** Statistical significance at 1% level.

## DISCUSSION

To the best of our knowledge, this is the first report on trust in physicians and satisfaction with the health system among Chinese smokers. The current study found that, in general, current smokers had a low level of trust in physicians. Current smokers would be more likely to rate trust in physicians lower than never smokers. Trust in physicians is the optimistic acceptance of a vulnerable situation in which the patient believes that the physician will act in the patient’s best interest^19^. Previous studies indicated that health issues, functional decline, and premature mortality are strongly related to smoking^20,21^. Considering the profound vulnerability created by smoking-related disease and mortality, the greater the sense of vulnerability, the greater the potential for either trust or distrust^19^. Smoking is associated with higher healthcare utilization by current smokers than those who never smoked^22,23^. Smoking patients may be more likely to stay with the practice and continue to see the same physician and thus have more continuity. Sufficient continuity with the same physician to allow for the establishment of a positive relationship between patient and physician, may help to improve trust^24^. However, profound vulnerability may also give rise to distrust in physicians^25,26^. Smoking is linked with worse health conditions^2^. Persons in the worse health conditions are more likely to have negative experiences in healthcare and be less satisfied with it^11^. Alternatively, worse health conditions may cause depressive symptoms or other negative feelings that cloud evaluations of trust in physicians^19^. Moreover, this study found that more cigarette smoking was associated with lower trust in physicians. Ever smokers with more pack-years have increased incidence of smoking-related disease^27^. More pack-years come with a greater sense of vulnerability and, therefore, more cigarette smoking creates higher distrust.

The present study also found that current smokers were less likely to rate greater satisfaction with the health system than never smokers. Individuals assess the health system on the basis of health outcomes. When the health system underperforms to produce inadequate health outcomes for whatever reason, satisfaction will decrease^28^. Previous studies have reported that current smokers are more likely to report their health as poor or very poor than never smokers^21,29,30^. Individuals who report being in poor or very poor health are less likely to be satisfied with the health system^31^.

Trust in physicians is decisive in patients’ willingness to seek care, reveal sensitive information, submit to treatment, adhere to treatment plans, remain with a physician, and recommend physicians to others^19,32^. There are more than 800 smoking cessation clinics in China. However, 5.6% of current smokers planned to quit smoking within one month in 2018, and only 1–2 patients per week per hospital sought assistance from these cessation clinics^1,33^. Lack of knowledge about the health hazards of tobacco and the absence of physician’s advice on smoking cession contribute to the low cessation rates in China^33^. Patients with higher trust in physicians are significantly more likely to understand smoking-related health problems and engage in smoking cessation^24^. This result may have significant implications for regaining trust in physicians by smokers. A trusting relationship between physician and smoker should be established before introducing smoking-related health problems and smoking cessation advice.

Trust looks forward, an expectation of future behavior, while satisfaction looks backward grounded on past experience^24^. Satisfaction with the health system has identified ways to improve health, reduce costs, and implement reform^21^. Current smokers had a relatively low level of satisfaction with the health system, and this result may have important implications for supporting health-system reform for tobacco treatment. First, the Chinese government should integrate tobacco dependence diagnosis, treatment, and monitoring, into the primary health system. Second, the medical insurance plans in China should consider covering smoking cessation treatment.

### Limitations

Our study has some limitations. First, self-reported smoking status has been used in this study, and thus shares the limitations of all self-reported data: recall bias and unreliability of responses under pressure. Moreover, self-reported daily and occasional smoking data are unavailable in the CFPS. This study used pack-years to measure an individual’s exposure to tobacco. Furthermore, we could not exclude ex-smokers who quit smoking in the past 30 days from the group of current smokers. Second, the question concerning trust in physicians or satisfaction with the health system was in our study a single question on an 11-point Likert scale. If respondents lack knowledge about the trust or satisfaction question, the responses may be inaccurate. Third, although the current study adjusted for a wide variety of control variables, it is possible that unknown or unmeasured confounders may explain the current findings.

## CONCLUSIONS

Current smokers had a low level of trust in physicians and low satisfaction with the health system. Current smokers were less likely to have a higher trust in physicians and satisfaction with the health system than never smokers. These results may have important implications for regaining trust in physicians from smokers and supporting health-system reform for tobacco treatment.
